# Protection of protease-activated receptor 2 mediated vasodilatation against angiotensin II-induced vascular dysfunction in mice

**DOI:** 10.1186/1471-2210-11-10

**Published:** 2011-09-28

**Authors:** Elizabeth Chia, Satomi Kagota, Enoka P Wijekoon, John J McGuire

**Affiliations:** 1 Cardiovascular Research Group, Division of BioMedical Sciences, Faculty of Medicine, Memorial University, St. John's, Newfoundland and Labrador, Canada; 2 Department of Pharmacology, School of Pharmaceutical Sciences, Mukogawa Women's University, Nishinomiya, Japan; 3 Department of Molecular and Cellular Biology, University of Guelph, Ontario, Canada

## Abstract

**Background:**

Under conditions of cardiovascular dysfunction, protease-activated receptor 2 (PAR2) agonists maintain vasodilatation activity, which has been attributed to increased cyclooxygenase-2, nitric oxide synthase and calcium-activated potassium channel (SK3.1) activities. Protease-activated receptor 2 agonist mediated vasodilatation is unknown under conditions of dysfunction caused by angiotensin II. The main purpose of our study was to determine whether PAR2-induced vasodilatation of resistance arteries was attenuated by prolonged angiotensin II treatment in mice. We compared the vasodilatation of resistance-type arteries (mesenteric) from angiotensin II-treated PAR2 wild-type mice (WT) induced by PAR2 agonist 2-furoyl-LIGRLO-amide (2fly) to the responses obtained in controls (saline treatment). We also investigated arterial vasodilatation in angiotensin II-treated PAR2 deficient (PAR2^-/-^) mice.

**Results:**

2fly-induced relaxations of untreated arteries from angiotensin II-treated WT were not different than saline-treated WT. Treatment of arteries with nitric oxide synthase inhibitor and SK3.1 inhibitor (L-NAME + TRAM-34) blocked 2fly in angiotensin II-treated WT. Protein and mRNA expression of cyclooxygenase-1 and -2 were increased, and cyclooxygenase activity increased the sensitivity of arteries to 2fly in only angiotensin II-treated WT. These protective vasodilatation mechanisms were selective for 2fly compared with acetylcholine- and nitroprusside-induced relaxations which were attenuated by angiotensin II; PAR2^-/- ^were protected against this attenuation of nitroprusside.

**Conclusions:**

PAR2-mediated vasodilatation of resistance type arteries is protected against the negative effects of angiotensin II-induced vascular dysfunction in mice. In conditions of endothelial dysfunction, angiotensin II induction of cyclooxygenases increases sensitivity to PAR2 agonist and the preserved vasodilatation mechanism involves activation of SK3.1.

## Background

The attenuation of endothelium-dependent vasodilatation elicited by hormones or shear stress is a condition observed in cardiovascular diseases. This condition is often referred to as endothelial dysfunction. It is thought that endothelial dysfunction is an early development in the time course of cardiovascular diseases. Clinical tests of patients for endothelial dysfunction include measuring the vasodilator responses of blood vessels to an agonist of the endothelium e.g. cholinergic agonist acetylcholine. Protease-activated receptor 2 (PAR2) is a G protein coupled receptor which can be activated by trypsin-like serine proteases and PAR2-activating peptides (PAR2-AP [1,2]. These peptides activate the endothelium to cause acute vasodilatation, decrease blood pressures and protect tissues from ischemia [3-5]. Studies in genetic hypertension, stroke and diabetes have reported that PAR2-AP vasodilatations were persistent despite endothelial dysfunction being present [6-10]. The acute mechanisms of action of PAR2-AP require further study because these pathways represent elements of vascular smooth muscle relaxation which are protected against the negative effects of cardiovascular diseases.

Under normal conditions PAR2-AP mediate acute vasodilatation of small caliber resistance arteries via nitric oxide and Ca^2+^-activated K^+ ^channels (K_Ca_) [11,12]. There are a few variations in the mechanisms that have been attributed to the PAR2-AP mediated vasodilatation mechanisms during endothelial dysfunction. These mechanisms have included the selective activation of SK3.1 [6,10], endothelial nitric oxide synthase (eNOS) [8], and cyclooxygenases (COX) [9]. In non-obese diabetes models of endothelial dysfunction, increased PAR2 expression was reported [8,9]. The uncertainty in the mechanisms of PAR2 vasodilatation in endothelial dysfunction may be due to the choice of experimental models particularly the reliance on genetic strains of rodents. Human diseases represent complex phenotypes so it is important to investigate PAR2 in multiple experimental models.

Chronic angiotensin II (ANG II) infusion produces a model of acquired hypertension and endothelial dysfunction in animals. It is also linked to pro-inflammation signaling pathways involving p38 mitogen activated protein kinase and nuclear factor-κB. These transcription pathways are proposed to partly link ANG II receptor signalling to changes in endothelial cell phenotype, which include induction of cyclooxygenase (COX-1 and COX-2). In endothelial cell culture conditions the p38 mitogen activated protein kinase and nuclear factor-κB pathways link cytokines to induction of PAR2 expression and are activated by PAR2-AP [13,14]. Interestingly, the induction of COX-2 in endothelial cells in culture enabled PAR2 to stimulate cells to produce PGI_2_, which was proposed as being vasculoprotective [13].

To date the effect of chronic ANG II-induced endothelial dysfunction on PAR2-AP vasodilatation is unknown. We have described a trend for higher blood pressures in PAR2 gene (*par2*) knockout mice (PAR2^-/-^) after two weeks of ANG II infusion [15]. These results may be consistent with the proposal of PAR2-mediated protection of blood vessels against the negative effects of chronic ANG II. The primary goal of this study was to determine the effects of ANG II-induced endothelial dysfunction on vasodilatation by PAR2-AP [2-furoyl-LIGRLO-amide, 2fly, [3]]. We also tested whether *par2 *gene deficiency was protective against chronic ANG II-induced endothelial dysfunction in the vasculature. Our findings provide new and significant additions to understanding vascular pharmacology and important interactions with cardiovascular pathology. Also, these findings highlight the potential for SK3.1 to be a potential pharmaceutical target for hypertension.

## Results

### Preserved PAR2-mediated relaxations of mesenteric arteries in ANG II C57

**Figure 1:**
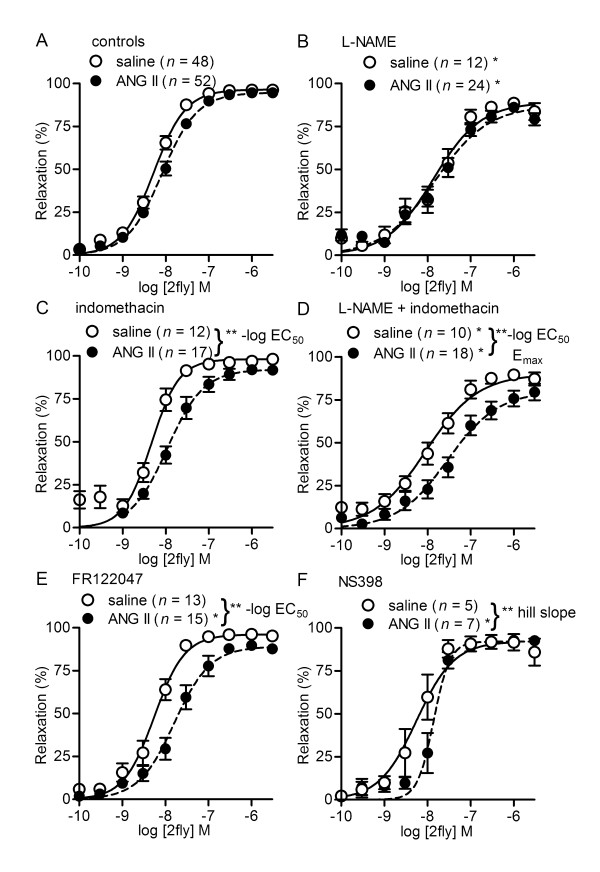
**NOS and COX inhibitor effects on PAR2 agonist-induced vasodilations in saline- and ANG II-treated C57**. C57BL/6J (C57) mice were pretreated with saline or angiotensin II (ANG II) for two weeks prior to experiments. Values are the mean ± SE (*n *= number of mice) for 2-furoyl-LIGRLO-amide (2fly)-induced relaxations of second order mesenteric arteries contracted submaximally by cirazoline. Arteries were exposed to inhibitors [100 μM L-NAME, 10 μM indomethacin, 100 μM L-NAME + 10 μM indomethacin, 3 μM NS398, 1 μM FR122047] for 20 min prior to cirazoline. *P < 0.05, inhibitor treatment compared with controls (A, untreated) within same pump treatment in (B) E_max_, -log EC_50 _[both pumps], hill slope [saline], (D) E_max _[both pumps], -log EC_50 _[ANG II], (E) -log EC_50_, and (F) hill slope. ** *P *< 0.05, inhibitor treatment in ANG II vs. saline in (C) -log EC_50_, (D) -log EC_50_, E_max_, (E) -log EC_50 _and (F) hill slope. Two-way ANOVA (pump treatment × inhibitors) followed by Bonferroni *post hoc *test.

To determine whether chronic ANG II treatment attenuated the vasodilator effectiveness of PAR2, we measured relaxations by the PAR2 activating peptide 2fly of mesenteric arteries contracted submaximally by α_1_-adrenoceptor agonist (cirazoline). Chronic ANG II did not reduce the effectiveness of PAR2 to cause vasodilatation of arteries (Figure 1A). 2fly-mediated relaxations of arteries of ANGII C57 vs. saline C57 were the same (Figure 1A, Table 1). These data indicated that PAR2-mediated vasodilatation by 2fly was protected against treatment by chronic ANG II. As we have reported elsewhere [3,10] PAR2 was confirmed as being the specific target of 2fly in the bioassay by our finding that concentrations up to 3 μM of this peptide had no effect on arteries from saline PAR2^-/- ^and ANG II PAR2^-/- ^(*P *> 0.05, relaxations not different than 0, data not shown).

**Table 1:** 2-fuoryl-LIGRLO-amide (2fly) concentration-response curves of mesenteric arteries from saline- and angiotensin II-treated C57 mice.

Treatment	*n*	-log EC_50_ (M)	E_max_ (%)	Hill slope	*n*	-log EC_50_ (M) ^a d^	E_max_ (%) ^b d^	Hill slope ^c f^
	*Saline*				*Angiotensin II*			
controls	48	8.3(0.1)	97(1)	2.1(0.1)	52	8.1(0.1)	96(1)	1.8(0.1)
L-NAME	12	7.8(0.2)^i^	90(2)^j^	1.9(0.6)*^j^*	24	7.8(0.1) ^j^	85(2)^i^	1.3(0.2)
indomethacin	12	8.4(0.1)	96(1)	2.1(0.5)	17	7.9(0.1)*^h^*	93(1)	1.5(0.2)
L-NAME + indomethacin	10	8.0(0.1)	90(2)^k^	1.2(0.3)	18	7.4(0.1)^g i^	81(4)^g i^	1.3(0.2)
NS398 3 μM	5	8.2(0.4)	92(4)	2.3(0.3)	7	7.9(0.1)	93(2)	4.7(1.6)^h i^
FR122047	12	8.3(0.1)	96(1)	2.0(0.3)	15	7.8(0.1)^h k^	91(2)	1.7(0.3)
NS398 + FR122047	5	8.0(0.2)	92(2)	0.9(0.1)	8	8.0(0.2)	94(2)	2.4(0.4)
CAY10441	7	8.3(0.2)	96(2)	1.8(0.3)	7	8.0(0.1)	96(1)	2.2(0.4)
AH6809 + L798106 + L161982	6	8.5(0.3)	97(1)	1.5(0.3)	6	8.0(0.1)	96(1)	2.2(0.4)
SQ29548	4	8.5(0.2)	96(1)	0.8(0.2)	6	7.9(0.2)	93(2)	2.0(0.4)
NS398 0.3 μM	7	8.1(0.1)	98(1)	2.1(0.3)	6	7.8(0.1)	95(1)	2.4(0.6)

Values are mean(SE), *n *= number of mice. Variables were determined by curve fitting data points from cumulative drug concentration-responses relationships to a four parameter logistic function. Treatments of arteries included antagonists of COX-1 (1 μM FR122047), COX-2 (0.3, 3 μM NS398), COX-1/2 (10μM indomethacin), NOS (100 μM L-NAME), prostaglandin E__2__receptors (1 μM AH6809, 1 μM L798106, 0.1 μM L161982), prostaglandin I__2__receptor (0.1 μM CAY10441) and thromboxane A__2__receptor (1 μM SQ29548). Comparisons were made by two-way ANOVA (pump × artery treatment) [^a^*P *< 0.0001, ^b^*P *< 0.01, and ^c^*P *< 0.05, effect of pump treatment, ^d^*P *< 0.0001, and ^e^*P *< 0.0005, effect of artery treatment, ^f^*P *< 0.01, interaction between pump and treatment] followed by Bonferroni *post hoc *tests: ^g^*P *< 0.01, and ^h^*P *< 0.05, compared to same inhibitor treatment in saline pump group [horizontal]; ^i^*P *< 0.001, ^j^*P *< 0.01, and ^k^*P *< 0.05, controls compared to inhibitor within same pump treatment [vertical].

C57, C57BL/6J; E_max_, maximum relaxation response where 100% is complete reversal of contraction.

### Contributions of NOS to preserved PAR2-mediated relaxations of mesenteric arteries in ANG II C57

In normal (*par2 *wild-type) mice it is well-established that eNOS contributes by a small extent to PAR2-mediated vasodilatation of small mesenteric arteries [3,6,10-12]. To determine whether an increased contribution by NOS to PAR2 activity could be attributed to the preserved vasodilator effectiveness of PAR2, we compared 2fly-induced relaxations in the absence (Figure 1A) and presence of L-NAME (Figure 1B). Treatment of arteries with L-NAME inhibited 2fly-induced relaxations compared with controls to the same extents in saline and ANG II C57 (Table 1). These data indicated there was not a significant change in endothelium-derived NO elicited by 2fly in ANG II C57.

### Contributions of COX to preserved PAR2-mediated relaxations of mesenteric arteries in ANG II C57

Inhibitors of COX have no effect on PAR2-mediated vasodilatations of C57 mesenteric arteries [10-12]. However, chronic *in vivo *treatment with ANG II has been reported to induce expression of cyclooxygenases in vascular tissues. To determine whether *de novo *contribution of COX to PAR2 activity could be attributed to preserved vasodilator effectiveness of PAR2, we compared 2fly-induced relaxations in the absence (Figure 1A) and presence of COX inhibitors (Figure 1C-F). As expected, the 2fly CRC of untreated arteries from saline C57 (Figure 1A, saline) were not different in the presence of COX inhibitors (Figure 1C-F, saline). The potency of 2fly in ANG II C57 (Figure 1A, ANG II) was reduced by either nonselective or selective COX isoform inhibitors (Figure 1C-F, ANG II). Indomethacin (nonselective COX inhibitor) in the absence (Figure 1C) and presence of L-NAME (Figure 1D) caused rightward shifts of the 2fly CRC in ANG II vs. saline C57. Similarly, COX-1 inhibitor (FR122047) rightward shifted the 2fly CRC in ANG II C57 vs. saline C57. COX-2 inhibitor (NS398) reduced the steepness (hill slope) of the 2fly CRC relationship in ANG II C57 vs. saline C57 (Figure 1F) and thus, inhibited the effectiveness of 2fly at the low to middle range of concentrations (0.1 nM to 10 nM).

To investigate whether a single metabolite of COX activity could be attributed to the potency changes for 2fly, we compared responses in the absence and presence of various receptor antagonists for prostanoids and thromboxane A_2_. The results of these experiments (bottom half of Table 1) did not support a role for any specific metabolite in the actions of COX inhibitors on 2fly potency in ANG II C57. 2fly CRC in ANG II C57 vs. saline C57 (Table 1) were not different in the presence of antagonists for PGI_2 _receptor (CAY10441), PGE_2 _receptors (AH6809, L798106, L161982), and thromboxane A_2 _receptor (SQ29548).

### Contributions of SK3.1 to preserved PAR-mediated relaxations of mesenteric arteries in ANG II C57

**Figure 2:**
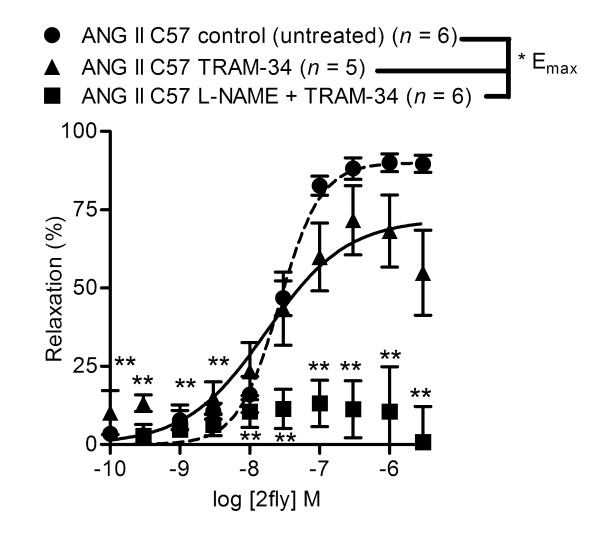
**Inhibition of PAR2 agonist-induced vasodilations in ANG II C57 by pretreatment with SK3.1 inhibitor**. C57BL/6J (C57) mice were pretreated with angiotensin II (ANG II) for two weeks prior to experiments. Values are the mean ± SE (*n *= number of mice) for 2-furoyl-LIGRLO-amide 2fly-induced relaxations of ANG II C57 second order mesenteric arteries contracted submaximally by cirazoline. Arteries were exposed to inhibitors [10 μM TRAM-34, 100 μM L-NAME + 10 μM TRAM-34] for 20 min prior to cirazoline. *P < 0.05, E_max_, 10 μM TRAM-34 and 100 μM L-NAME + 10 μM TRAM-34 compared to untreated (control) arteries, one-way ANOVA followed by Bonferroni *post hoc *test. **P > 0.05, L-NAME + TRAM-34, relaxations at each dose of 2fly compared to 0, one-sample *t*-test.

It has been demonstrated numerous times [6,11,12] that combined inhibition of NOS, SK3.1, and SK2.2/2.3 is required to block vasodilatation of normal mouse mesenteric arteries by PAR2-activating peptides; and acetylcholine. Recent work by us [10] indicated a primary role for SK3.1 in contributing to preserved PAR2 vasodilatation in obese diabetic mouse mesenteric arteries. To determine the contribution of SK3.1 to the vasodilatation mediated by PAR2 in ANG II C57, we investigated 2fly-induced relaxations in the presence of specific SK3.1 inhibitor TRAM-34, which was done in the absence and presence of NOS inhibitor L-NAME. Indeed treatment of arteries by SK3.1 inhibitor reduced the effectiveness of 2fly in ANG II mice (Figure 2) and when combined with pretreatment by L-NAME the 2fly CRC in ANG II C57 was blocked compared with controls (Figure 2).

### ACh mediated vasodilatations in saline vs. ANG II and C57 vs. PAR2^-/-^

**Figure 3:**
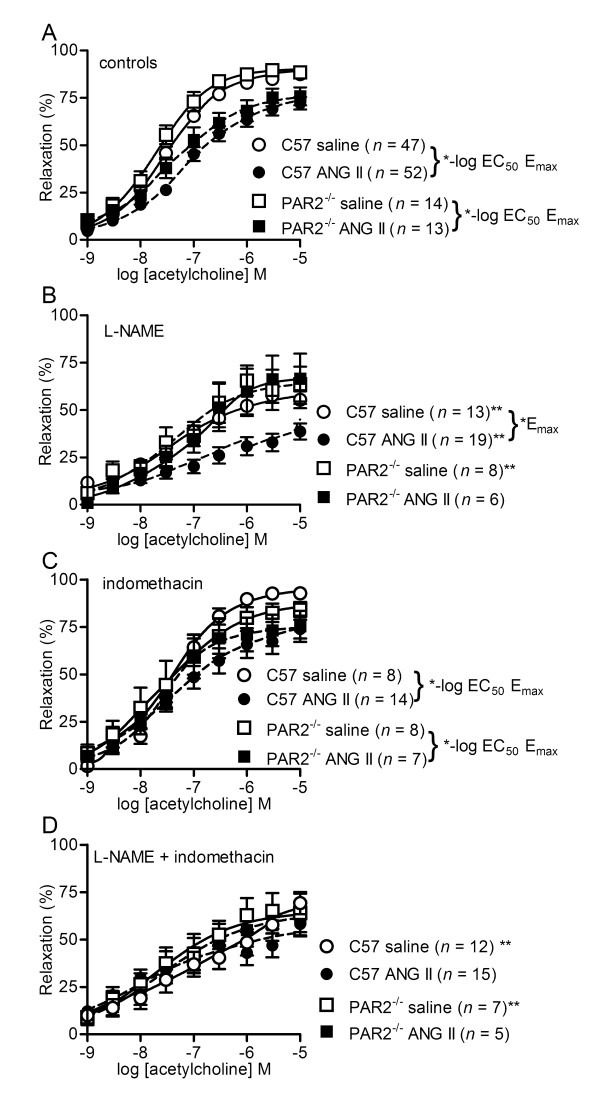
**Acetylcholine-induced vasodilatations in C57 and PAR2^-/- ^mice administered saline and ANG II**. C57BL/6J (C57) and protease-activated receptor 2 gene knockout (PAR2^-/-^) mice were pretreated with saline or angiotensin II (ANG II) for two weeks prior to experiments. Values are the mean ± SE (*n *= number of mice) for ACh-induced relaxations of second order mesenteric arteries contracted submaximally by cirazoline. Arteries were exposed to inhibitors [100 μM L-NAME, 10 μM indomethacin, 100 μM L-NAME + 10 μM indomethacin] for 20 min prior to cirazoline. *P < 0.05, saline vs. ANG II in (A, C) -log EC_50 _and E_max _and (B) E_max_. **P < 0.05, inhibitor treatment compared with controls (A, untreated) within same pump treatment. Comparisons by two-way ANOVA (inhibitor treatments × pump) followed by Bonferroni *post hoc *test.

We determined ACh mediated vasodilatations of arteries from saline and ANG II treatments of both C57 and PAR2^-/- ^to reproduce previous reports of the negative effects of ANG II treatment on endothelial cell function. These experiments also assessed the protection attributed to PAR2^-/- ^genotype against the negative effects of ANG II. PAR2^-/- ^genotype did not afford protection for untreated arteries against the negative effects on endothelial function of ANG II treatment (Figure 3A (controls), Table 2). Vasodilatation by ACh of untreated (control) arteries was decreased in ANG II vs. saline treatments to the same extent in C57 vs. PAR2^-/- ^(Figure 3A, Table 2).

**Table 2:** Acetylcholine concentration-response curves of mesenteric arteries from saline- and angiotensin II-treated mice.

Treatment ^i^	Strain	*n*	-log EC_50_ (M)	E_max_ (%)	Hill slope	*n*	-log EC_50_ (M) ^a^	E_max_ (%) ^b c^	Hill slope
	*Saline*				*Angiotensin II*				
Controls	C57	47	7.5(0.1)	90(1)	1.0(0.1)	52	7.0(0.1)	78(3)^d^	1.0(0.1)
	PAR2^-/-^	14	7.7(0.1)	91(2)	1.0(0.1)	13	7.5(0.3)	77(5)	0.9(0.2)
L-NAME	C57	13	7.5(0.2)	63(5)^e f^	0.7(0.1)	19	6.8(0.3)	49(5)^g h^	0.7(0.1)
	PAR2^-/-^	8	7.2(0.3)	67(8)^e f^	0.7(0.1)	6	7.4(0.3)	68(13)	1.1(0.2)
indomethacin	C57	8	7.4(0.2)	96(2)	1.5(0.6)	14	7.3(0.2)	81(4)	0.8(0.1)
	PAR2^-/-^	8	7.5(0.3)	87(3)	1.0(0.1)	7	7.6(0.1)	76(8)	0.9(0.2)
L-NAME + indomethacin	C57	12	7.3(0.3)	70(6)^e f^	0.8(0.2)	15	7.6(0.4)	59(6)^g h^	0.6(0.1)
	PAR2^-/-^	7	7.7(0.2)	69(10)^e^	0.7(0.1)	5	7.4(0.2)	66(8)	0.6(0.1)
FR122047	C57	7	7.5(0.1)	87(3)	1.1(0.1)	11	6.9(0.3)	71(6)	1.5(0.8)
NS398	C57	8	7.7(0.3)	89(3)	1.3(0.3)	10	7.2(0.3)	78(8)	0.7(0.1)
FR122047 + NS398	C57	3	7.5(0.3)	75(4)	1.0(0.5)	7	7.4(0.2)	74(8)	1.3(0.3)
AH6809 + L798106 + L161982	C57	6	7.5(0.2)	90(3)	1.2(0.2)	6	6.8(0.2)	91(3)	1.2(0.3)
CAY10441	C57	7	7.4(0.2)	88(3)	1.0(0.1)	7	6.8(0.3)	86(3)	1.1(0.3)

Values are mean (SE), *n *= number of mice. Variables were determined by curve fitting data points from cumulative drug concentration-responses relationships to a four parameter logistic function. Treatments of arteries included antagonists of COX-1 (1 μM FR122047), COX-2 (0.3, 3 μM NS398), COX-1/2 (10 μM indomethacin), NOS (100 μM L-NAME), prostaglandin E__2__receptors (1 μM AH6809, 1 μM L798106, 0.1 μM L161982), prostaglandin I__2__receptor (0.1 μM CAY10441). Comparisons were made by two-way ANOVA (pump × artery treatment) [^a^*P *< 0.05, ^b^*P *< 0.0001, effect of pump (saline vs. angiotensin II), ^c^*P *< 0.001, effect of artery treatments] followed by Bonferroni *post hoc *tests: ^d^*P *< 0.05, ANG II C57 controls compared to saline C57 controls; ^e^*P *< 0.05, inhibitor group compared with saline C57 controls; ^f^*P *< 0.05, inhibitor group compared with saline PAR2^-/- ^controls; ^g^*P *< 0.05, group compared with ANG II C57 controls; ^h^*P *< 0.05, inhibitor group compared with ANG II PAR2^-/- ^controls; ^i ^data from PAR2^-/- ^were included as artery treatment factor for analyses.

C57, C57BL/6J; E_max_, maximum relaxation response where 100% is complete reversal of contraction; PAR2^-/-^, protease-activated receptor 2 gene knockout mice.

We determined ACh mediated vasodilatations in the absence and presence of L-NAME in C57 and PAR2^-/- ^to investigate the involvement of eNOS in these responses. While eNOS contribution to ACh-mediated vasodilatation was the same in saline C57 vs. saline PAR2^-/- ^(Figure 3B), there was less eNOS contribution to ACh mediated relaxations in ANG II C57 vs. ANG II PAR2^-/- ^(Figure 3B). This was found in the observation that L-NAME caused no further attenuation of ACh relaxations in ANG II PAR2^-/- ^(Figure 3B) compared with untreated arteries of ANG II PAR2^-/- ^(Figure 3A, Table 2).

We tested ACh-mediated vasodilatations in the absence and presence of COX inhibitors to determine the selectivity of their actions on the potency of endothelium-dependent agonists in ANG II C57. These experiments indicated that the COX inhibitors effects in ANG II C57 were affecting selectively 2fly in our bioassays. Treatments of arteries by indomethacin in the absence (Figure 3C) or presence of L-NAME (Figure 3D) compared with controls (Figure 3A) did not differentially affect ACh CRC in saline- vs. ANG II-treated mice. In addition, ACh CRC were not significantly affected by selective antagonists of COX-1, COX-2, PGI_2 _receptor, and PGE_2 _receptors (Table 2).

We determined the effects of combined inhibition of eNOS, sGC, SK2.3, SK2.3, and SK3.1 to confirm the involvement of these elements in the residual ACh-mediated vasodilatations. A combination of L-NAME, ODQ, apamin plus TRAM-34 was sufficient to block ACh CRC (*P *> 0.05, not different than 0, *n *= 3 in saline C57, ANG II C57, saline PAR2^-/-^, ANG II PAR2^-/-^, data not shown).

### Nitroprusside relaxations of arteries

**Figure 4:**
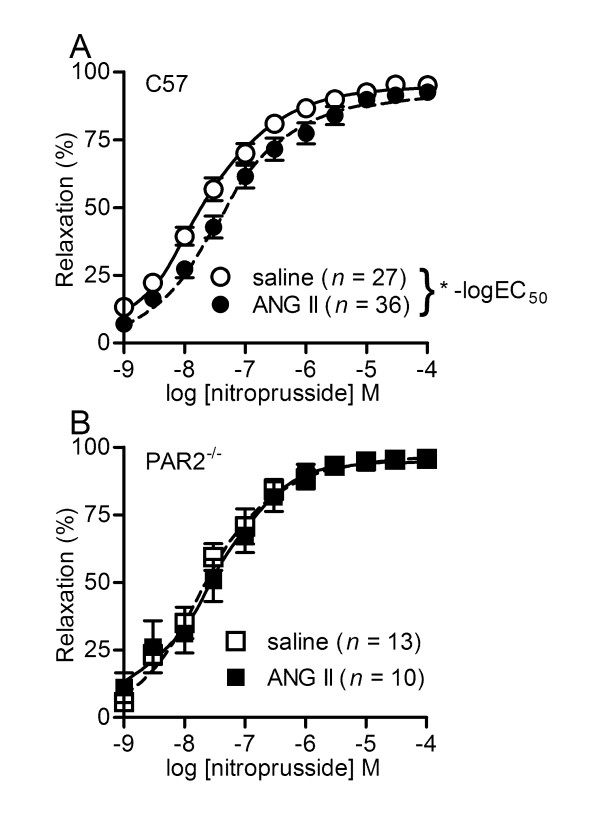
**Nitroprusside-induced vasodilatations in C57 and PAR2^-/- ^mice administered saline and ANG II**. C57BL/6J (C57) and protease-activated receptor 2 gene knockout (PAR2^-/-^) mice were pretreated with saline or angiotensin II (ANG II) for two weeks prior to experiments. Values are the mean ± SE (*n *= number of mice) for nitroprusside-induced relaxations of second order mesenteric arteries contracted submaximally by cirazoline. Arteries were exposed to inhibitors for 20 min prior to contraction by cirazoline and the cumulative addition of nitroprusside. Relaxation (%) represents the reversal of the contraction. *P < 0.05, saline C57 vs. ANG II C57 in -log EC_50_. Comparisons by two-way ANOVA (pump × inhibitors) followed by Bonferroni *post hoc *test.

To test the sensitivity of vascular smooth muscle to NO, nitroprusside-induced vasodilatations were determined. Indeed nitroprusside, which spontaneously releases NO and bypasses the endothelium, was slightly less potent in ANG II C57 vs. saline C57 (Figure 4A). The nitroprusside CRC was shifted to the right in ANG II C57 vs. saline C57 (Figure 4A, Table 3). Surprisingly, we found that PAR2 ^-/- ^genotype was protective against the potency shift caused by ANGII. The nitroprusside CRC were not different in ANG II PAR2^-/- ^vs. saline PAR2^-/- ^(Figure 4B, Table 3).

**Table 3:** Nitroprusside concentration-response curves of mesenteric arteries from saline- and angiotensin II-treated mice.

Treatments	Strain	*n*	-logEC_50_ (M)	E_max_ (%)	Hill slope	*n*	-logEC_50_ (M) ^a^	E_max_ (%)	Hill slope
		*Saline*				*Angiotensin II*			
Control	C57	27	7.7(0.1)	95(1)	0.9(0.1)	36	7.4(0.1)^b^	94(2)	1.2(0.1)
control ^c^	PAR2^-/-^	13	7.6(0.2)	96(1)	0.9(0.3)	10	7.7(0.2)	96(1)	1.0(0.3)
NS398	C57	7	7.8(0.3)	94(2)	1.3(0.4)	11	7.3(0.2)	95(1)	1.7(0.4)
FR122047	C57	9	7.7(0.2)	95(3)	1.1(0.2)	11	7.2(0.2)	94(2)	1.3(0.3)
AH6809 + L798106 + L161982	C57	5	7.4(0.2)	95(2)	1.3(0.5)	4	7.9(0.1)	96(1)	2.2(1.0)
CAY10441	C57	6	7.9(0.2)	96(1)	1.2(0.4)	5	7.4(0.3)	96(1)	1.7(0.8)

Values are mean (SE), *n *= number of mice. Variables were determined by curve fitting data points from cumulative drug concentration-responses relationships to a four parameter logistic equation. Treatments of arteries included antagonists of COX-1 (1 μM FR122047), COX-2 (3 μM NS398), prostaglandin E_2 _receptors (1 μM AH6809, 1 μM L798106, 0.1 μM L161982), prostaglandin I_2 _receptor (0.1 μM CAY10441). Comparisons were made by two-way ANOVA (pump × artery treatments) [^a^*P *< 0.05, effect of pump (saline vs. angiotensin II)] followed by Bonferroni *post hoc *tests: ^b^*P *< 0.05, control compared to saline pump treatment; ^c^data of control arteries from PAR2^-/- ^were included as a treatment factor for analyses.

C57, C57BL/6J mice; E_max_, maximum relaxation response where 100% is complete reversal of contraction; PAR2^-/-^, protease-activated receptor 2 gene knockout mice.

To test for potential off-target effects of the various inhibitors of COX on vascular smooth muscle sensitivity to NO, nitroprusside-induced vasodilatations in the presence and absence of these inhibitors were determined. The various inhibitors of COX and PGI_2_/PGE_2 _receptors did not affect vascular smooth muscle sensitivity to NO of the arteries. Nitroprusside CRC were not affected by the presence of FR122047, NS398, CAY10441 and the combination of AH6809, L798106 and L161982 (Table 3).

### Endothelium-dependence of vasodilators in saline C57 and ANG II C57 arteries

To determine whether ACh- or 2fly-induced relaxations were due to direct activation of vascular smooth muscle cells, the inner linings of mesenteric arteries were damaged and then the effectiveness of the agonists were assayed. As expected from previous work in various mouse arteries [6,10,12], in both saline and ANG II C57 the ACh- and 2fly-induced relaxations were reduced to effective 0 after being subjected to endothelium removal, but nitroprusside relaxed these arteries [relaxation (%), saline (*n *= 5)/ANG II (*n *= 4): ACh: 8 ± 5/3 ± 3; 2fly: 2 ± 2/8 ± 6; nitroprusside: 98 ± 2/96 ± 3].

### Contraction of arteries by cirazoline in PAR2^-/- ^vs. C57

We determined the contractions of arteries by α_1_- adrenergic receptor agonist cirazoline after saline and ANG II treatment of C57 and PAR2^-/- ^to investigate the effect of PAR2^-/- ^genotype on vasoconstrictor agonist effectiveness. These experiments also investigated the effects of ANG II on arteries responses to cirazoline. Surprisingly, cirazoline was a more potent vasoconstrictor in PAR2^-/- ^than in C57. ANG II treatments did not increase the vasoconstrictor effectiveness of cirazoline in arteries of either strain. In saline PAR2^-/- ^(*n *= 11) and ANG II PAR2^-/- ^(*n *= 12) the cirazoline CRC were shifted to the left (more potent) of saline C57 (*n *= 27) and ANG II C57 (*n *= 33), respectively [***P < 0.0005, two way ANOVA effect of strain on -logEC_50_: saline PAR2^-/-^, 7.7 ± 0.1; ANG II PAR2^-/-^, 7.7 ± 0.1; saline C57, 7.4 ± 0.1; ANG II C57, 7.4 ± 0.1].

### PAR2-AP 2fly and acetylcholine under baseline conditions in C57 and PAR2^-/-^

To determine whether the chronic ANG II treatment induced a potential for vasoconstrictor activity by 2fly and ACh, we measured isometric tension of mesenteric arteries after exposing tissues to agonists. We found no evidence of vasoconstrictor activity for these agonists in the mesenteric arteries. There were no observed changes in tension at baseline after the addition of either 2fly (up to 10 μM) or acetylcholine (up to 300 μM) to arteries from saline C57 (*n *= 3), ANG II C57 (*n *= 3), saline PAR2^-/- ^(*n *= 3) and ANG II PAR2^-/- ^(*n *= 3).

### mRNA expression of COX-1, COX-2, and PAR2

To determine whether mRNA expression of COX-1, COX-2 and PAR2 were increased by ANG II treatment of mice, we measured the mRNA expression pattern of these genes by real-time PCR. COX-2 mRNA expression (normalised to triad gene reference) was significantly upregulated in ANG II C57 (Table 4). COX-1 and PAR2 mRNA expression (normalised to triad gene reference) were not significantly different between treatments. Given the effectiveness of 2fly was reduced by COX inhibitors in ANG II C57 (Figure 1C-F) and these PAR2 mediated relaxations were endothelium-dependent, we also normalized the mRNA for COX-1 and COX-2 relative to *par2 *reference to relate mRNA expression to the functional observations. According to this analyses, COX-1 and COX-2 mRNA (normalised to *par2*) were significantly upregulated in ANG II C57 (Table 4).

**Table 4:** Protease-activated receptor 2, cyclooxygenase-1 and cyclooxygenase-2 mRNA expression in angiotensin II- and saline-treated mice.

mRNA	Ratio (angiotensin II to saline)			
	normalised to triad		normalised to *par2*	
protease-activated receptor 2	0.6	0.2-1.8		
cyclooxygenase-1	1.5	0.5-4.5	2.4^b^	1.2-4.2
cyclooxygenase-2	2.9^a^	0.6-16	4.5^c^	0.9-26

mRNA expression in mesenteric arterial cascades from mice was determined by real-time PCR and normalised to triad housekeeping, and to protease-activated receptor 2 genes (*par2*).

angiotensin II (ANG II) C57, *n *= 12; saline C57, *n *= 11.

Values reported are the mean and SE range (mean minus lower SE and mean plus upper SE).

^a^*P *< 0.05, ^b^*P *< 0.001, ^c^*P *< 0.0005, gene upregulated in ANG II C57 compared with saline C57. See Methods for details of statistical analyses.

To determine whether the PAR2^-/- ^genotype affected the constitutive expression of COX-1/2 we compared the gene expression pattern in C57 with PAR2^-/-^. COX-1 and COX-2 mRNA were not significantly different between PAR2^-/- ^and C57 [ratio PAR2^-/- ^(*n *= 4) to C57 (*n *= 4), *P *> 0.05, COX-1, 1.1 (0.7 - 1.4); COX-2, 1.1 (0.7 - 1.8)].

### Protein expression of COX isoforms in mesenteric arterial beds

**Figure 5:**
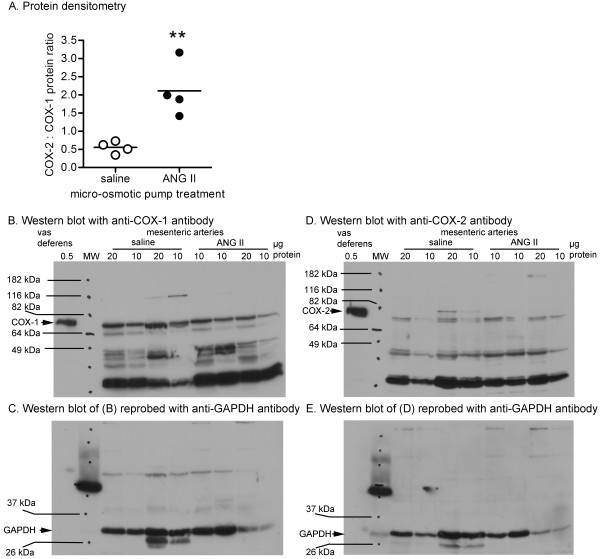
**Expression of COX-1 and COX-2 in saline C57 and ANG II C57 mesenteric arteries**. C57BL/6J (C57) mice were pretreated with saline or angiotensin II (ANG II) for two weeks prior to experiments. Upper panel (A) summarises protein densitometry data collected from four saline C57 and four ANG II C57 for COX-1 and COX-2. Protein expression ratios (COX-2 to COX-1) were determined from the target bands normalised to the densities of GAPDH. **P < 0.005, saline vs. ANG II, Student's *t*-test for unpaired data. Lower panels (B - E) show representative data collected from two saline C57 and two ANG II C57 mice indicating immunoreactive bands corresponding to (B) COX-1, (D) COX-2, and after stripping and re-probing membranes, the bands corresponding to (C, E) GAPDH. Each sample was assayed in duplicate at the protein amounts indicated in each lane. Internal reference protein (vas deferens) and relative molecular weight ladder (MW) are also shown. Pen marks indicate the location of prestained MW, which were transcribed by overlay of films on membranes.

To determine whether upregulated COX mRNA expression patterns and effectiveness of COX inhibitors were matched to increased levels of COX proteins in arteries, we measured the levels of COX proteins by Western blot. The ratio of COX-2 to COX-1 protein expression in mesenteric arterial beds was significantly higher in ANG II C57 vs. saline C57 (Figure 5).

## Discussion

Our main finding was the PAR2-AP vasodilatation of small caliber arteries was protected against the negative effects of chronic ANG II infusion. Activation of SK3.1 was critical to the mechanism of PAR2 mediated relaxations in ANG II mice. ANG II infusion in mice attenuated NO-dependent relaxations by ACh and increased the ratio of COX-2 to COX-1 protein expression. Inhibiting either COX-1 or COX-2 decreased the potency of 2fly in ANG II-treated mice. ANG II treatment did not increase PAR2 expression in arteries. The PAR2-AP-mediated acute vasodilatation during ANG II infusion involves components of smooth muscle relaxation pathways that are resistant to the negative effects of endothelial dysfunction in cardiovascular disease. These components may be potential therapeutic targets for protection against the consequences of cardiovascular disease.

Inhibition of SK3.1 and endothelial NO synthase was sufficient to block PAR2-AP mediated vasodilatation. However, we found that chronic ANG II infusion of mice also led to a change in PAR2 mediated relaxations of vascular smooth muscle from indomethacin-insensitive to indomethacin-sensitive. These finding suggest COX is part of a minor supplementary mechanism of action for PAR2 after ANG II treatment. In human volunteers, aspirin treatment decreased the PAR2-AP vasodilatation of forearm blood vessels [16]. In non-obese diabetic mice, a selective COX-2 inhibitor decreased the effectiveness of PAR2-AP on aortas [9]. This attenuation of PAR2-AP became larger as the effectiveness of ACh decreased and the mice developed hyperglycemia with age [9]. Under cell culture conditions, exposure to PAR2 agonists increase COX-2 mRNA, protein and PGI_2 _[13,17] in endothelial cells. We found either FR122047 (selective COX-1 inhibitor) or NS398 (selective COX-2 inhibitor) was sufficient to decrease the potency of 2fly in ANG II-treated mice. Antagonists of PGI_2 _and PGE_2 _receptors in our study did not replicate the effects of COX inhibitors. Therefore, it appeared that PGI_2 _and PGE_2 _receptors were not involved with the supplementary mechanism.

We speculate that PAR2 activates COX-dependent production of vasodilator reactive oxygen species, which would be independent of prostaglandin receptors. In our study, ANG II infusion reduced the potency of nitroprusside in arteries. Other studies have attributed the attenuation of nitroprusside to quenching of the NO by free radicals. In human vein endothelial cells, PAR2-AP increased reactive oxygen species production from mitochondrial complex III and lesser amounts from COX-2 [18]. It has been reported that COX-dependent reactive oxygen species [19] could activate big K_Ca _which is expressed on vascular smooth muscle. This would be consistent with our interpretation that COX inhibitors had an indirect effect on the PAR2 relaxations.

Our study highlights that the experimental model of cardiovascular disease impacts the mechanisms of PAR2 action in blood vessels [6,7,20]. Albeit our study focused on a small caliber artery which is used to model the resistance vasculature and thus, uses less NO than large caliber conductance type (e.g. femoral arteries, aortas) for endothelium-dependent relaxations by PAR2 [12,21]. In other genetic hypertension models, the PAR2 vasodilatation of small caliber arteries was not linked to COX [7,20]. Also, our current and previous studies indicate that persistent PAR2 vasodilatation is entirely endothelium-dependent. However, in non-obese diabetic mice aortas and spontaneously hypertensive rat basilar arteries the PAR2-AP vasodilatations were endothelium-independent [9,22]. On the other hand, the central role of SK3.1 has been highlighted as a common finding in all of our studies on small caliber arteries. We reported that in obese diabetic mice that SK3.1 was the key link in the preserved PAR2 mediated vasodilation [10]. Recent studies have demonstrated the utility of targeting SK2.2, SK2.3 and SK3.1 agonists for modulating vascular tone, presumably by endothelium-dependent hyperpolarization, and lowering blood pressure in ANG II-induced hypertensive mice [23]. The protection of the PAR2 vasodilatation against ANG II-acquired hypertension suggests that endothelial K_Ca _activation by selected membrane receptors could also provide the vasculature with the ability to compensate or replace NO during disease. Some investigators have reported that uncoupled endothelial NO synthase may contribute to a superoxide-mediated vasodilatation of aortas during endothelial dysfunction in diabetes, which was also proposed to be linked to ANG II [24]. However, inhibition of uncoupled endothelial NO synthase by L-NAME remains a point of controversy [25,26] so it is uncertain whether a similar connection between PAR2 and uncoupled endothelial NO synthase exists in the ANG II infusion model.

The results of experiments in PAR2^-/- ^indicate that *par2 *null genotype had mixed effects on the negative outcomes of ANG II infusions on blood vessels. First, there was an increased sensitivity of arteries to adrenergic receptor stimulation in PAR2^-/-^. Second, there was less L-NAME sensitive endothelial NO-derived relaxation activity (ACh-induced) in PAR2^-/- ^after treatment with ANG II. Third, the potency of nitroprusside in ANG II PAR2^-/- ^was not decreased as in ANG II C57. The first of the effects above would be expected to increase systolic and pulse pressures in PAR2^-/-^. The second and third effects mentioned could be interpreted as protective compensatory mechanisms that may limit increases in blood pressures in PAR2^-/-^. Our radiotelemetry study of blood pressures indicated that by the end of treatment period, ANG II produced larger systolic and pulse pressure changes in PAR2^-/- ^than in C57 [15]. A limitation to the scope of our study was the focus on disruption by ANG II of endothelium-dependent vasodilatation. We examined only one vasoconstrictor system, necessarily to establish conditions for bioassay, so it was possible that PAR2^-/- ^may have affected vasoconstrictor systems other than α_1 _adrenergic receptors e.g. 5-hydroxytryptamine, thromboxane A_2 _[27]. In the past 10 years, the literature weighs heavily with evidence linking activated PAR2 to pro-inflammatory signal transduction in endothelial cells [28]. There is also a broad literature contending that chronic elevated levels of ANG II elicits increased pro-inflammatory signalling by cytokines and pro-thrombotic conditions that contribute to the disruption of normal endothelial and vascular smooth muscle cell biology [29]. In wild-type animals, it is possible that increased activity of coagulation factors and or other serine proteinases during chronic ANG II infusion leads to PAR2 activation and thus, the production of cytokines that cause vascular dysfunction. Accordingly, it would be expected that the production of pro-inflammatory mediators elicited by ANG II in *par2 *null mice may be attenuated and thus, the disruptions by ANG II of the endothelium and vascular smooth muscle are reduced.

## Conclusions

PAR2-AP effectiveness is protected against the negative effects of ANG II. *par2 *null expression had mixed effects *in vitro *on the negative outcomes of ANG II hypertension. The preserved PAR2 vasodilatation which is mediated largely via SK3.1 may lead to discovery of other endothelial cell signaling that is resistant to vascular diseases.

## Methods

### Animals

Control (*par2 *wild-type) mice (C57BL/6J, C57) and *par2 *gene deficient mice (PAR2^-/-^; B6.Cg-*F2rl1*^tm1mslb^/J) were purchased from the Jackson Laboratories (Bar Harbor, ME). Genotypes of PAR2^-/- ^were confirmed by PCR using specific primer sets as per supplier's protocol [30]. Male mice (10-30 weeks of age; 25-32 g) were fed a standard regular salt feed (NIH-31 autoclavable open formula mouse diet; Zeigler Bros Inc., Gardners, PA, USA) and provided water *ad libitum *while housed individually in air filter-topped cages in a room of the Animal Care Facility. All protocols were approved by the Institutional Animal Care Committee of Memorial University in accordance with the guidelines and principles for use of animals in research by the Canadian Council on Animal Care.

### Sources of drugs and reagents for myograph studies

Unless stated otherwise, all drugs and reagents were obtained from Sigma Aldrich (Oakville, Ontario, Canada). Other sources included: Tocris BioScience (Ellisville, MO, USA), FR122047, CAY10441, AH6809, L798106, L161982, NS398, SQ29548; Toronto Research Chemicals (Toronto, Ontario, Canada), TRAM-34; Peptides International (Louisville, KT, USA), 2-furoyl-leu-ile-gly-arg-leu-orn-amide (2fly). Stock solutions of indomethacin, TRAM-34, ODQ, FR122047, CAY10441, AH6809, L798106, L161982, NS398 and SQ29548 were made in dimethylsulfoxide and added as a 1/1000 dilution to tissue bath solutions. Stock solutions of all other drugs were made in water.

### Chronic ANG II infusion

On day 0 of the ANG II infusion protocol, a micro-osmotic pump (ALZET model 1002, Durect Corp., Cupertino, CA, USA) containing either isotonic saline (0.25 μl/h) or ANG II (1 μg Ile^5^-angiotensin kg^-1 ^min^-1^) was implanted dorsally subcutaneously. Mice underwent surgeries to implant the micro-osmotic pumps (~10-15 min) using isoflurane/oxygen anesthesia, followed by monitoring their recovery for 4-6 h before being returned to their home cages. Twelve to 14 days later mice were anaesthetised with isoflurane and then killed by cervical dislocation. In a subset of these mice, blood pressures were recorded by radiotelemetry methods which we reported previously [15].

### Relaxation bioassays

To test the effect of chronic ANG II on endothelial function and the influence of PAR2^-/-^, the vasodilatation of small caliber mesenteric arteries from ANG II-treated mice were compared to the responses in saline-treated mice. Twelve to 14 days after implanting a micro-osmotic pump, a mouse was killed and their mesenteric arcades with attached adipose, blood vessels and nerves were dissected free from gastrointestinal tract in situ and immediately place in ice-cooled Krebs buffered bicarbonate solution (114 mM NaCl, 4.7 KCl, 0.8 mM KH_2_PO_4_, 1.2 mM MgCl_2_, 2.5 mM CaCl_2_, 25 mM NaHCO_3 _and 11 mM D-glucose). Rings of second order mesenteric arteries (2-12 per mouse) of 1-2 mm lengths were isolated from arcades and positioned in small wire myograph chambers (DMT 610 M, DMT 620 M) for measurement and recording of isometric tension as described [6,10]. Each chamber contained Krebs buffered solution (pH 7.4) bubbled with 95% O_2_/5% CO_2 _at 37°C. Initial resting tension for each artery was determined as described [6], with initial effective pressure at 7.98 kPa. After an initial equilibration period (1 h), vascular reactivity was measured. Tissues were deemed viable when the addition of 90 mM potassium chloride produced a tension change > 1 mN/mm length. In artery rings from each of the four treatment groups (saline C57, saline PAR2^-/-^, ANG II C57, ANG II PAR2^-/-^), contractions were determined from cumulative additions of cirazoline (1 nM-3 μM) to the baths. To determine the relaxation response, the rings were contracted by addition of small increments of cirazoline to submaximal tensions [50-80% of E_max_], even in the presence of inhibitors, based on concentration-response curves (CRC) data. After a stable contraction was obtained, a PAR2-activating peptide, 2fly (0.1 nM-3 μM) [3], ACh (1 nM-10 μM) or nitroprusside (0.1 nM-100 μM) was added cumulatively to the bath. The order of vasodilator agonist addition was determined in a randomly assigned order. The contraction and relaxation responses were measured in either the absence or presence of inhibitors (20 min), which included N^G^-nitro-L-arginine methyl ester (L-NAME, NOS inhibitor, 100 μM), indomethacin (COX-1/2 inhibitor, 10 μM), ODQ (soluble guanylyl cyclase (sGC) inhibitor, 1 μM), apamin (SK2.2/2.3 inhibitor, 1 μM), TRAM-34 (SK3.1 inhibitor, 10 μM), NS398 (COX-2 inhibitor, 0.3, 3 μM), FR122047 (COX-1 inhibitor, 1 μM), CAY10441 (PGI_2 _receptor antagonist, 0.1 μM), PGE_2 _receptors antagonists combination [AH6809 (1 μM), L798106 (1 μM), L161982 (0.1 μM)], and SQ29548 (thromboxane A_2 _receptor antagonist, 1 μM). Inhibitor treatment with these antagonists were reported as being effective [6,31-37]. Endothelium-dependence of relaxation by the test agonists was determined by pulling a human hair through the lumen of arteries and then assaying the agonists [10].

### mRNA expression

mRNA expression was assayed in mesenteric arterial arcades using quantitative real-time PCR methods as described [10]. Extraction and purification of total RNA from frozen tissues followed the manufacturer's instructions (Qiagen, Mississauga, Ontario, Canada). TaqMan RNA-to-CT 1-step kit procedures were followed according for real-time measurement of target gene expression on an ABI Prism 7000 light cycler (Applied Biosystems, Foster City, CA, USA). For each target gene primer-fluorescent probe set the amount of RNA used was optimized. Gene specific primer-fluorescent probe sets were available commercially (Applied Biosystems, assay/product ID: COX-1, Mm00477214_m1; COX-2, Mm00478374_m1; PAR2, Mm00433160_m1; glyceraldehyde 3-phospate dehydrogenase, GAPDH, 4352932E; β_2_-microglobulin, Mm00437762_m1; calnexin, Mm00500330_m1; Streetsville, Ontario, Canada). The primer-fluorescent probe sets were validated for *par2 *and the other genes, previously [10]. In some instances to accommodate low individual yield of samples, RNA was pooled from two to three mice and each pooled set was treated as one independent sample. Each sample was measured in duplicate. A triad reference gene expression approach (GAPDH, β_2_-microglobulin, calnexin) was used from normalization of sample material, and the efficiencies for primer sets for each were included in all calculations.

### Protein expression in whole mesenteric arteries

Western blots were performed on mesenteric arterial protein following the same procedures as described [10]. To accommodate low individual yield of arterial protein from each mouse, protein was pooled from up to three mice to obtain each independent tested sample. Membranes were probed separately with specific primary antibodies against COX-1 (#160109, Cayman Chemicals, Ann Arbor, MI, USA), COX-2 (SC-1745, Santa Cruz Biotechnology, Santa Cruz, CA, USA) and GAPDH (SC-25778, Santa Cruz Biotechnology). As positive controls for both COX-1 and COX-2 detection, 500 ng of total protein isolated from mouse vas deferens was loaded in separate wells [38]. Immunoreactive bands on membranes were visualized on x-ray film by chemiluminescence detection according to supplier (Super-Signal West Pico, Thermo Fisher Scientific Inc., Rockford, IL, USA). Image J (National Institute of Health, Bethesda, MD, USA) software was used to quantify target band intensities. Representative immunoblots of experiments were repeated independently three times.

### Data Analyses

Individual CRCs were analysed by nonlinear regression curve fitting of drug concentration-relaxation/contraction response relationships using a four parameter logistic function. We compared the variables of negative log EC_50_, Hill slope and E_max _values between groups by one-way or two-way (pump × artery treatment) ANOVA as indicated in the legends of Figures and Tables. Statistics indicating significant main effects or interactions were followed by Bonferroni *post hoc *for multiple comparison testing. As indicated in legends to Figures, the data points at each cumulative drug concentration were compared to a hypothetical value of 0 by one-sample *t*-test when data points from individual CRC did not fit a logistic function (R^2 ^< 0.8). *P < 0.05 was considered significant. Myograph data were reported as mean ± SE, and *n *= number of mice. For protein expression data, target band density in each lane was normalised to the corresponding density for GAPDH then the COX-2 to COX-1 ratio for each sample was determined. Protein data are reported as mean ± SE, and *n *= number of independent samples. Comparisons of COX-2 to COX-1 ratio were made by Student's *t*-test for unpaired data. *P < 0.05 was considered significant. In mRNA expression experiments, group-wise comparison of relative expressions and statistical analyses of the relative expression results from real-time PCR were done using REST 2008 software [39] with data combined from 1 to 4 independent experimental runs. Data are reported as the mean and SE interval for the ratio of ANG II to saline normalised target genes expression where *n *= number of independent samples. *P < 0.05 was considered significant.

## List of abbreviations

ANG II: angiotensin II; 2fly: 2-furoyl-LIGRLO-amide; COX: COX-1/2: cyclooxygenase-1/2; CRC: concentration-response curve; eNOS: endothelial NOS; K_Ca_: Ca^2+^-activated potassium channel; L -NAME: N^g^-nitro-L-arginine methylester; PAR2: protease-activated receptor 2; *par2*: PAR2 gene in mice; PAR2^-/-^: *par2 *knockout mice; PAR2-AP: PAR2-activating peptides; SK2.2/2.3: small-conductance K_Ca_; SK3.1: intermediate-conductance K_Ca_; sGC: soluble GC.

## Authors' contributions

JJM and EC participated in research design. All authors conducted experiments and performed data analysis. All authors wrote or contributed to the writing of draft manuscripts. All authors have read and approved the final manuscript.
